# Mindscapes of Neurorehabilitation: Insights from the European Congress on Neurorehabilitation 2023 – Part II

**DOI:** 10.25122/jml-2023-1030

**Published:** 2023-11

**Authors:** Cristian Andriescu, Alexandra Gherman, Stefana-Andrada Dobran, Dafin Muresanu

**Affiliations:** 1 RoNeuro Institute for Neurological Research and Diagnostic, Cluj-Napoca, Romania; 2 Sociology Department, Babes-Bolyai University, Cluj-Napoca, Romania; 3 Department of Neuroscience, Iuliu Hatieganu University of Medicine and Pharmacy, Cluj-Napoca, Romania

The European Federation of Neurorehabilitation Societies organizes every other year ECNRs, events which focus on bringing together specialists from various fields of neurorehabilitation, i.e., scientists, clinicians, therapists, and patient organizations, as well as representatives of governmental organizations, to join forces and develop environments oriented toward patient care.

The scientific program of the 7^th^ European Congress of Neurorehabilitation included 13 workshops, seven plenary lectures, 28 symposiums (out of which 7 were in cooperation with partner societies), 121 invited speakers, 109 ePoster sessions, and two roundtables.

Part I of **Mindscapes of Neurorehabilitation: Insights from the European Congress on Neurorehabilitation** 2023 highlighted the importance of neurorehabilitation as a core focus element of EFNR and several partner societies, the significance of the Dysphagia Course Series (organized with ESO and the Stroke Alliance for Europe), at its second edition in Lyon, France and it also provided a first glimpse into the four-day hybrid event (August 30 - September 2), the ECNR 2023, organized in collaboration with The French Society of Physical and Rehabilitation Medicine (SOFMER).

**Figure 1:**
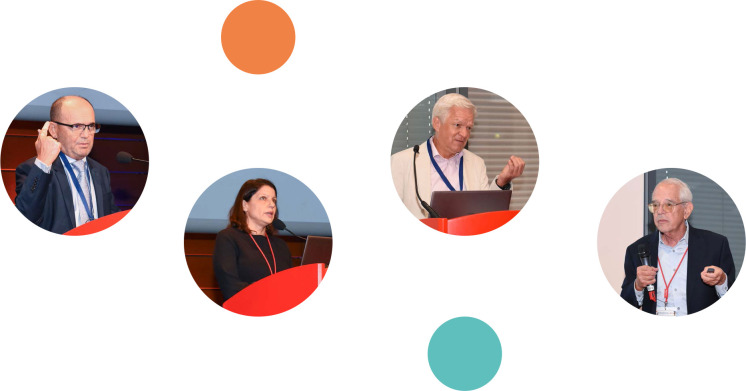
From left to right: Prof. Dafin Muresanu (Plenary Lecture); Dr. M. Trapl-Grundschober (Neurorecovery session); Prof. Christian Matula (Chairman of the AMN’s Education Committee); Prof. Michael Chopp (EFNR-AMN Session)

**Figure 2:**
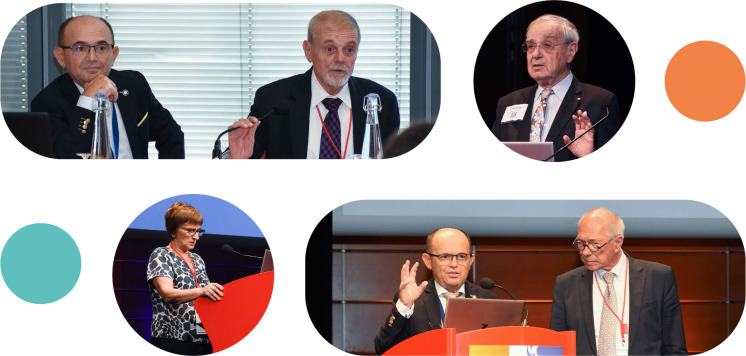
Prof. Dafin Muresanu and Prof. Natan Bornstein (EFNR-ESO EAST Joint Session) – upper left; Prof. Vladimir Hachinski (Closing Lecture) – upper right; Prof. Ljubica Konstantinovic (New Societies Session) – lower left; Prof. Dafin Muresanu and Prof. Volker Homberg (Congress Closing Remarks) – lower right

As previously stated, the event brought together close to 800 participants and speakers from 57 countries (Figure 1, Figure 2), and it was marked by an Opening Ceremony, hosted by Prof. Dafin Muresanu, President of EFNR, welcoming also numerous distinguished personalities to the podium (Prof. Volker Hömberg - WFNR President, Prof. Wolfgang Grisold – WFN President), Prof. Matilde Leonardi - Institutional delegate at WHO), Prof. Francesca Pezzella - Chairperson of SAP-E), Prof. Johannes Vester – President of AMN), Heinrich Binder – Honorary and Founding President of EFNR), and Isabelle Bonan (President of SOFMER), who all offered a warm address to the audience.

Shortly after the ceremony, the audience engaged in the first plenary lecture held by Prof. Isabelle Loubinoux (University of Toulouse, France) on stem cells and biomaterials transplantation in stroke and traumatic brain injury (TBI). Afterwards, Prof. John Krakauer from the University of Baltimore, USA, explained the complex composition of post-stroke arm paresis and how it might be reversed and, therefore, bringing a substantial contribution to improving the patient's quality of life. Continuing the presentations, Dr. Trapl-Grundschober discussed screening procedures for dysphagia in acute ischemic stroke, recovery mechanisms, possible complications, and the importance of early screening and guidelines. Professor Dafin Muresanu, EFNR president, delved into combination therapy for aphasia following stroke, with a focus on the importance of neurotrophic factors and multimodal drugs, such as Cerebrolysin, in this pathology. The results of the ESCAS Trial in Cluj-Napoca come to support the relevance of such pharmacological approaches. Dr. Andreas Winkler then offered the audience an insight into the topic of post-stroke hemiparesis and motor control, focusing on the four pillars of stroke rehabilitation (timing, therapy, intensity, and medication). Concluding the session, Dr. Massimo Marano focused on spasticity considering its high heterogeneity. He discussed approaches to treatment using Botulinum Toxin therapy, and provided a glimpse into the Comprehensive Observational and Longitudinal study on the Outbreak of Stroke related Spasticity focusing on the Early-onset Management with Botulinum toxin (COLLOSEO) trial in Italy.

The plenary sessions continued on 1^st^ September with the Presidential lecture where Prof. Dafin Muresanu provided a comprehensive overview of the current understanding of the concept of brain reserve. To begin with, he introduced the concept by discussing its definitions, theoretical framework, neurobiological basis, and clinical implications. The presentation then delved into the feasibility of quantifying brain reserve, highlighting the role of biomarkers in predicting and tailoring neurorecovery. A significant focus was placed on operationalizing brain reserve, emphasizing integrating and evaluating existing data to develop an individual resilience index (IRI). The potential to enhance or recover brain reserve was explored, focusing on the impact of new technologies on neurobiology and its subsequent clinical implications. The integration of brain reserve in clinical research was also discussed, suggesting that outcomes are influenced by the interaction between the intervention and the individual’s brain reserve.

Dr. Stefan Strilciuc launched a discussion on resilient health systems, focusing on the role of health policy and economics in neurorehabilitation. Discussing the CENTER-TBI study results, Dr. Strilciuc set the scene for fruitful discussions in the context of a roundtable, which addressed resource allocation, expenditures, and the association with patient outcomes.

Next, the current president of WFNR, Prof. Volker Hömberg, approached the present status of neurorehabilitation worldwide alongside possible developments that may lead to progress in the field. As one-third of people are currently living with a condition that benefits from rehabilitation, context-appropriate strategies are needed to ensure improved outcomes. Because neurorehabilitation extends beyond the bio-clinical concept to incorporate social sciences, clear strategies, considering both classical neurorehabilitation methods as well as high-tech options, should be at the forefront of political decisions.

Expanding the collaborative spirit of the EFNR, the Congress featured joint sessions with Partner Societies. The partnered sessions with the World Health Organization (WHO), the World Federation of Neurology (WFN), ESO, AMN, and the Society for the Study of Neuroprotection and Neuroplasticity (SSNN) fostered a global perspective on neurorehabilitation and underscored the interrelation of global efforts in advancing neurorehabilitation research, practice, and policy.

The EFNR - AMN joint session began with the presentation of Prof. Johannes Vester, the AMN President, on traumatic brain injury research. Prof. Vester discussed the importance of randomized clinical trials, focusing on scale selection for evaluation. Discussions on the CAPTAIN trials on moderate to severe brain injury highlighted the principle of multidimensional approaches. Prof. Christian Matula, then discussed the history of the CAPTAIN trials in the context of TBI as a worldwide pressing problem and complex affection, as well as the paradigm shift in TBI research. Further on, Prof. Bogdan Popescu approached the topic of recent developments in TBI treatment, focusing on the C-RETURN study, a scientific pursuit currently ongoing in Cluj-Napoca. Lastly, Professor Michael Chopp offered a glance into the role of nanoparticles in treating neurological disorders, focusing on their benefits, mechanisms, and applications.

The SSNN Session was opened by Distinguished Professor Vladimir Hachinski, who discussed brain circulation, advising for the monitoring of blood pressure following a stroke or head trauma and highlighting the interconnection of cerebrovascular and neurological systems. Further on, Prof. Muresanu informed the audience of the importance of targeting microcirculation to enhance post-stroke neurorecovery. After an insight into stroke therapies such as thrombolysis and thrombectomy, he presented some conclusions for future developments in acute ischemic stroke that target the protection of microcirculation and the blood-brain barrier. Prof. Muresanu discussed avenues for enhancing thrombolysis and pinpointed the role of neuroprotective agents as adjunct therapies. Lastly, Prof. Sasa Filipovic (Research Professor at University of Belgrade, Institute for Medical Research) lectured on using Transcranial Magnetic Stimulation (TMS) targeting, more particularly, the dorsolateral prefrontal cortex and the posterior parietal cortex in neurological affections. TMS has been used in several pathologies (e.g., Alzheimer’s disease, post-stroke cognitive impairment, Parkinson’s disease, and multiple sclerosis) with positive results on global cognition, language, memory, and psychological symptoms.

The Stroke Action Plan for Europe/ESO-EAST Session commenced with Prof. Francesca Pezzella addressing early rehabilitation in an acute stroke unit setting. Prof. Pezzela underscored the importance of clear guidelines and the advantages of stroke care units in reducing disability and achieving better patient outcomes. The Stroke Action Plan for Europe (SAP-E) aims to reduce the absolute number of strokes across the continent, ensure that almost all stroke patients are treated in dedicated facilities, develop national plans for stroke alongside the entire care chain, and fully implement national strategies for multisectoral public health interventions. Moving forward, Prof. Natan Bornstein (Chairman of the Israeli Stroke Society - ISS) approached the topic of post-stroke cognitive impairment, an often-overlooked outcome of stroke, and addressed the lack of expert consensus on diagnosing tools, the important clinical scales, and the pathway for treatment for this complex affection. Lastly, Axel Kohlmetz (member of the ESO-EAST Managerial Advisory Board) offered a glance at the implementation of the Stroke Action Plan in Europe to achieve its objectives and key indicators.

**Figure 3:**
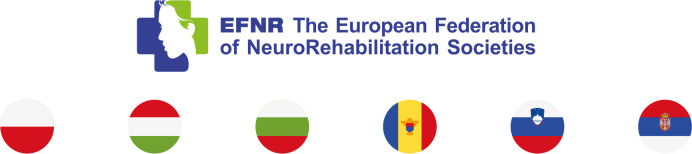
New neurorehabilitation societies enrolled in EFNR - Poland, Bulgaria, Hungary, the Republic of Moldova, Slovenia, and Serbia

The main goal of EFNR’s European strategy is to expand the institutional network. This pursuit commenced in early spring at Bistrita, Romania, where EFNR organized the first EFNR-WFNR Eastern European Regional Meeting. During this assembly, representatives from the national neurorehabilitation societies from Poland, Bulgaria, Hungary, the Republic of Moldova, Slovenia, and Serbia pledged their commitments to adhere to EFNR as new institutional members (Figure 3). Moving forward to Lyon, within the frame of the New Societies Session, Prof. Ljubica Konstantinovic (University of Medicine and Pharmacy, Belgrade, Serbia), Prof. Dimitar Maslarov (University of Medicine and Pharmacy, Sofia, Bulgaria), and Dr. Danu Glavan (University of Medicine Pharmacy, Chisinau, Republic of Moldova) offered a comprehensive lecture on the state of affairs on neurorehabilitation in their countries, highlighting the pressing need for funding and support from national authorities in order to keep pace with the developments in the field.

The Closing Lecture of the Congress had Distinguished Prof. Vladimir Hachinski at the stand discussing holistic brain health, an approach that integrates the cerebral, mental, and social components, all interlinked to one another. Professor Hachinski warned on the epidemic of loneliness and isolation, which increase the risk of disease, and discussed the importance of comprehensive evaluation and the components of holistic brain health, as well as its role in the prevention of the direst affections, such as stroke, dementia, and heart disease.

Prof. Dafin Muresanu and Prof. Volker Hömberg concluded the 7^th^ European Congress of Neurorehabilitation by extending their appreciation to the participants, speakers, organizers, partner societies, and industry, as well as a warm invitation to the upcoming World Congress on Neurorehabilitation, which will take place in Vancouver, Canada, on 22-25 May 2024.

The European Federation of Neurorehabilitation Societies has a consistent portfolio of ongoing projects. Stay in tune with developments in the field of neurorehabilitation by visiting our website, www.efnr.org.

